# IOL Tilt and Decentration Estimation from 3 Dimensional Reconstruction of OCT Image

**DOI:** 10.1371/journal.pone.0059109

**Published:** 2013-03-15

**Authors:** Xiaogang Wang, Jing Dong, Xiaoliang Wang, Qiang Wu

**Affiliations:** 1 Affiliated Sixth People's Hospital, Shanghai Jiao Tong University, Shanghai, P. R. China; 2 The First Hospital of Shanxi Medical University, Shanxi, P. R. China; 3 School of Aeronautics and Astronautics, Shanghai Jiao Tong University, Shanghai, P. R. China; National Eye Institute, United States of America

## Abstract

**Purpose:**

To evaluate intraocular lens (IOL) tilt and decentration by anterior segment optical coherence tomography (AS-OCT) using 3-dimensional (3D) reconstruction method.

**Design:**

Prospective observational case series.

**Participants:**

Thirty-nine patients (39 eyes) were included.

**Methods:**

The IOL positions of all eyes were examined by AS-OCT. Images were obtained in 4 axes (0–180 degrees, 45–225 degrees, 90–270 degrees, and 135–315 degrees) using the quadrant-scan model. The cross-sectional images were analyzed with MATLAB software.

**Main Outcome Measures:**

The angle (θ) between the reference pupillary plane and the IOL plane, the distances between the center points of the pupil circle and the IOL on the x-axis (dx) and y-axis (dy) and the spatial distance (ds) were calculated after 3D-reconstruction.

**Results:**

The mean angle (θ) between the pupillary plane and the IOL plane was 2.94±0.99 degrees. The mean IOL decentration of dx and dy was 0.32±0.26 mm and 0.40±0.27 mm, respectively. The ds of the IOL decentration was 0.56±0.31 mm. There was no significant correlation between the ocular residual astigmatism (ORA) and the tilted angle or the decentration distance. There was a significant correlation between the ORA and total astigmatism (r = 0.742, *P*<0.001). There was no significant correlation between the postoperative best corrected visual acuity (BCVA) and the ORA (r = 0.156; *P* = 0.344), total astigmatism (r = 0.012; *P* = 0.942), tilted angle (θ; r = 0.172; *P* = 0.295) or decentration distance (dx: r = 0.191, *P* = 0.244; dy: r = 0.253, *P* = 0.121; ds: r = 0.298, *P* = 0.065).

**Conclusions:**

AS-OCT can be used as an alternative for the analysis of IOL tilt and decentration using 3D-reconstruction.

## Introduction

The accurate alignment (tilt and decentration) of the intraocular lens (IOL) in the capsular bag is important for aspects of optical performance such as astigmatism, best-corrected visual acuity (BCVA) and high-order aberrations [1]–[4]. Several methods, such as Scheimpflug imaging, Purkinje reflection, ultrasound biomicroscopy, the anterior segment analysis system and photographic documentation, can be used for IOL position analysis in the clinic [5]–[9]. Anterior segment optical coherence tomography (AS-OCT) has been used to image the IOL position and to evaluate the postoperative IOL tilt in relation to the limbus in previous studies, but there are no reports of postoperative IOL decentration estimation with AS-OCT using a 3D-reconstruction method [10], [11]. In our study, we analyzed the decentration of IOLs implanted within the capsular bag after uneventful phacoemulsification using AS-OCT and correlated the results with BCVA and refractive outcomes.

## Materials and Methods

We used a single-piece, spherical, heparin surface-modified, foldable hydrophilic acrylic IOL (6-mm optic, 12.5-mm overall length; Hexavision, Paris, France). The pupils were not dilated, but 5 minutes of dark adaption were needed before image capture, and AS-OCT cross-sectional images were taken in the dark by the same technician using the Visante anterior segment OCT (Carl Zeiss Meditec, Dublin, California, USA) in the sitting position. This AS-OCT has an axial resolution of approximately 18 µm and a transverse resolution of approximately 60 µm with 1310-nm wavelength. With a scan speed of almost 8 frames/second and a 16×6 mm imaging range, the cornea, anterior chamber angle, iris, and part of posterior chamber lens were visualized. The anterior segment quadrant-scan model was used. The images were obtained in 4 axes with the same scan model (0–180 degrees, 45–225 degrees, 90–270 degrees, and 135–315 degrees), and the anterior and posterior optical surfaces of the IOL were imaged with reference to the position of the iris.

The eye plane image and two angle cross-sectional images (0–180 degrees, 90–270 degrees) were analyzed according to the method of Figure 1, 2, 3 and 4 by MatLab software version 7.8.0.347 (MathWorks) to complete the 3D-reconstruction of the IOL and the pupillary plane and then to calculate the IOL decentration and tilt angle.

**Figure 1:**
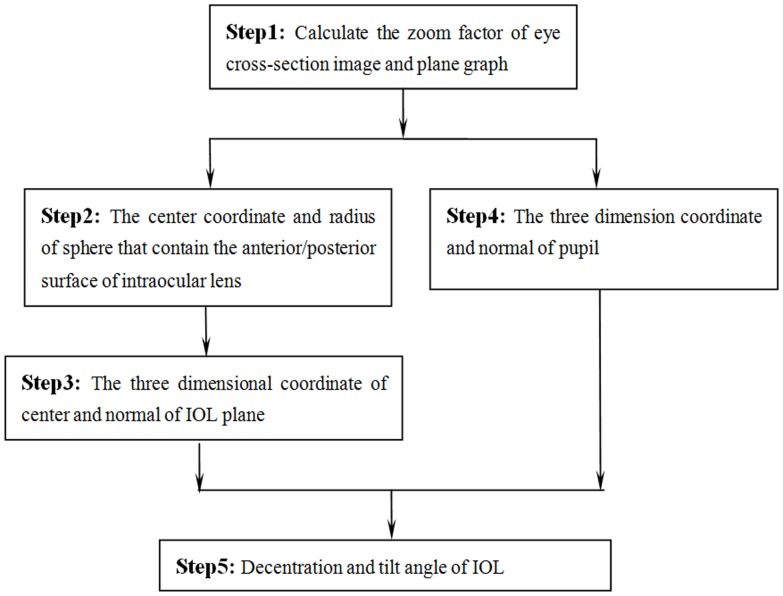
A flow chart depicting the decentration and tilt angle analysis of the intraocular lens (IOL).

**Figure 2:**
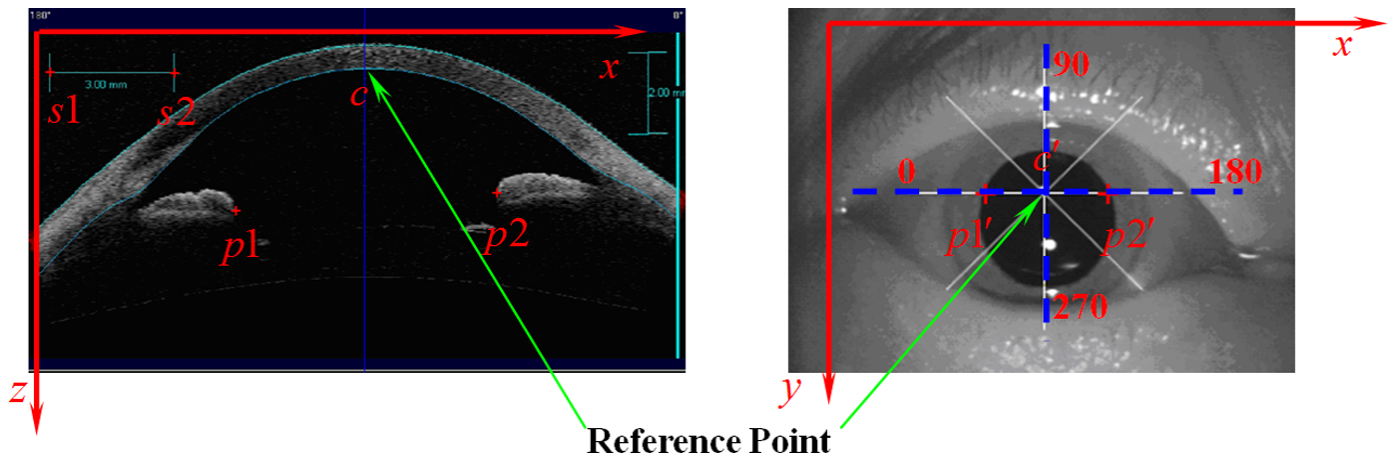
The diagrammatic presentation of reference frame and different point symbol.

**Figure 3:**
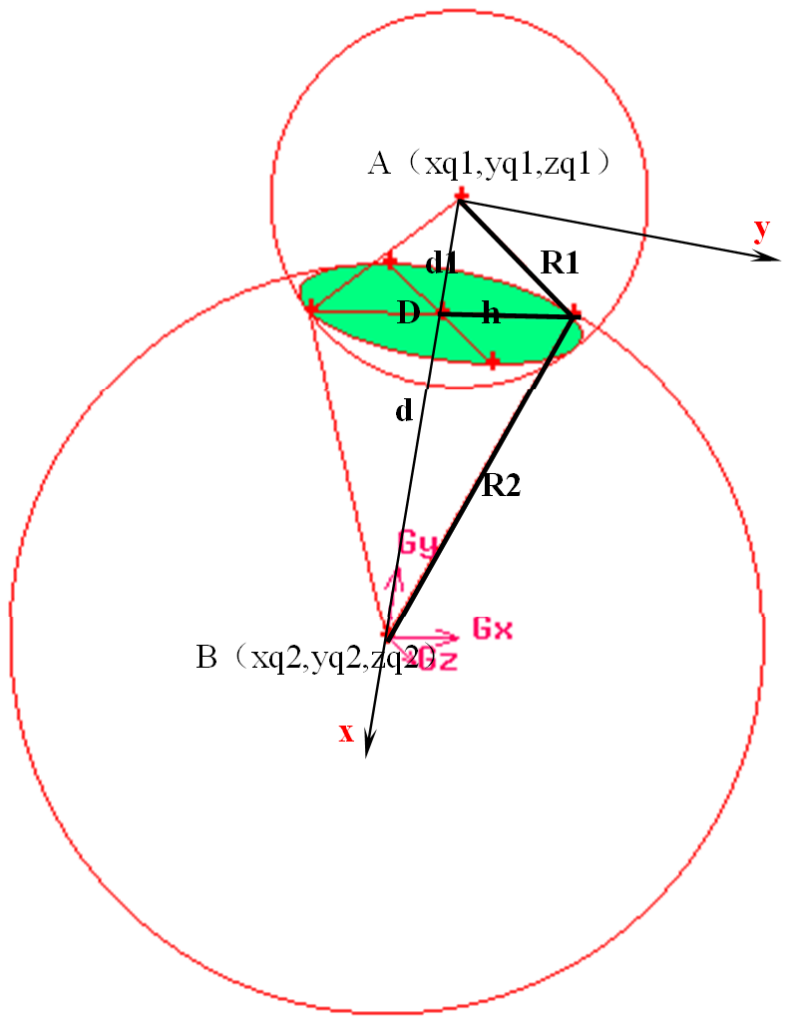
A sketch of the three-dimensional reconstruction about the IOL plane in the coordinates of (Gx, Gy, Gz). Sphere A with R1 as the radius contains the posterior surface of the IOL, and sphere B with R2 as the radius contains the anterior surface of the IOL. Green circle with h as the radius, which is the intersectant plane of sphere A and B, is the IOL plane.

**Figure 4:**
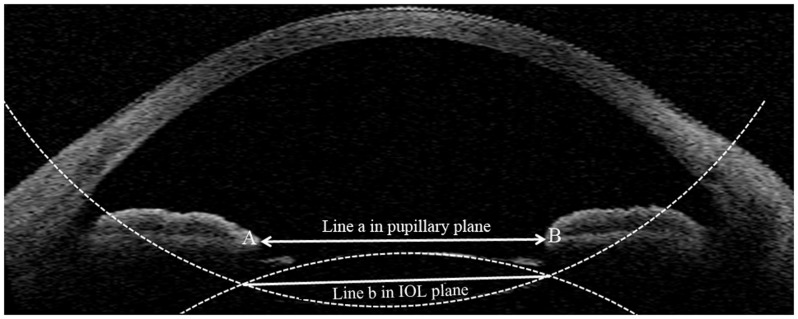
Anterior segment optical coherence tomography analysis of intraocular lens (IOL) tilt angle between the pupillary plane and the IOL plane. The pupillary plane was reconstructed using three different dots in two different scanning angle cross-sectional images as indicated by arrows A and B (the endpoint of the iris smooth muscle), and the IOL plane can be reconstructed using the 3D-reconstruction method. Line a was drawn in the pupillary plane, and line b was drawn in the IOL plane.

**Figure 5:**
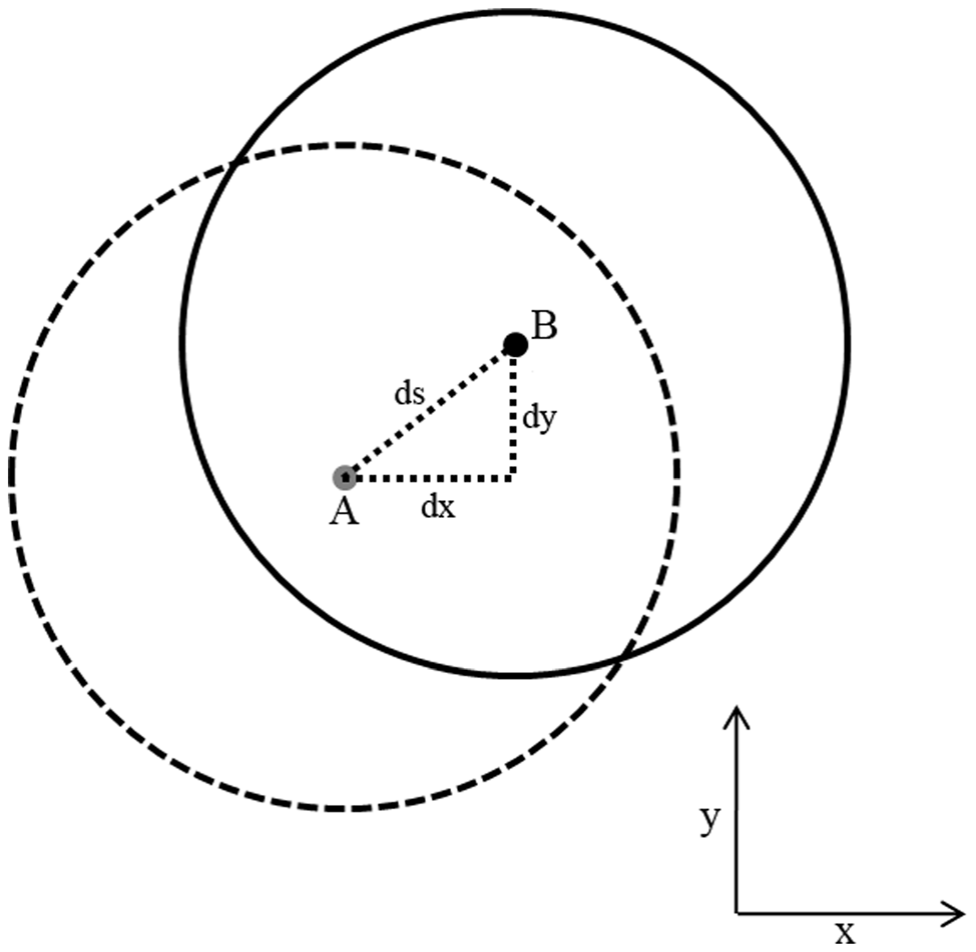
The sketch of the decentration distance from center A (pupillary center) to center B (IOL center). dx  =  decentration in x-axis; dy  =  decentration in y-axis; ds  =  spatial decentration.

When the reference pupillary plane and the IOL plane were parallel, the IOL was not considered to be tilted. The angle (θ) between the two planes was calculated. When the center points of the pupillary plane and the IOL were overlapping, the optic was not considered to be decentered. We only analyzed the decentration in the x-axis (dx) and the y-axis (dy) and the spatial distance (ds; Figure 5). Corneal topography was examined using Atlas (Carl Zeiss Meditec). All patients underwent retinoscopy and refraction, and BCVA was checked with Snellen distant vision acuity charts. Ocular residual astigmatism (ORA) was calculated by the method of Alpins and Goggin, and a graphical correlation was performed [12].

### AS-OCT imaging method

**Figure 6:**
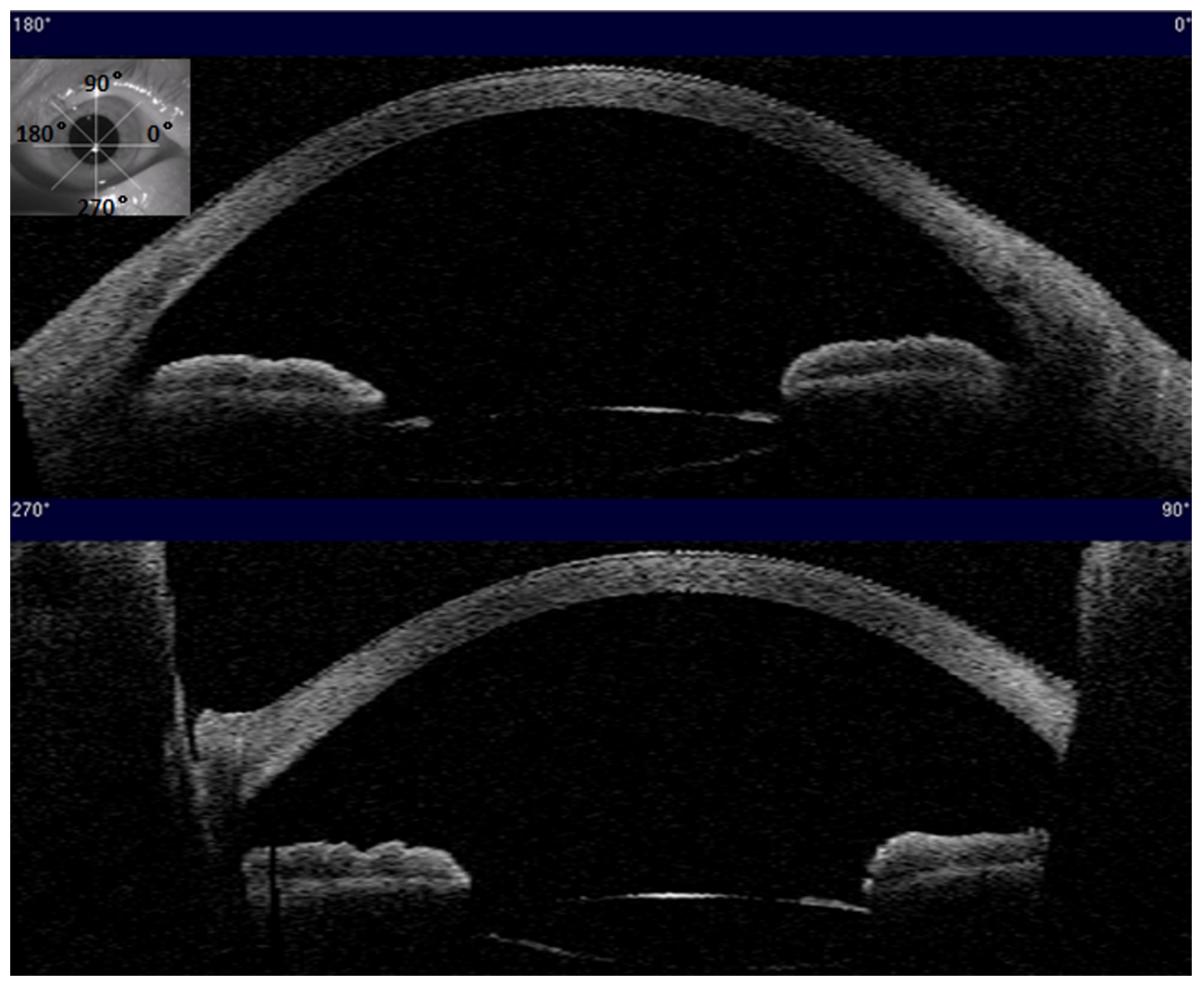
The eye plane image (top left corner) and cross-sectional images of a patient using the anterior segment quadrant-scan model.

Anterior segment OCT can provide cross-sectional images and eye plane images simultaneously (Figure 6). The tilt and decentration of the IOL can be obtained by 3D-reconstruction using these images. Under repeatability conditions, independent test results are obtained with the same method, on the same image, by the same operator and the repeatability was investigated in 3 eyes. We investigated repeatability by obtaining continuous 10-time measurements of tilted angle and decentration distance for each eye. All measurement was performed by the same operator. In this study, we investigated both intersession and interoperator reproducibility. Intersession reproducibility was investigated by acquiring values on two separate sessions by one single operator. To investigate interoperator reproducibility, two operators each obtained a set of values on every subject images during the same analysis session. Both intersession and interoperator reproducibility were investigated for a random group of 15 eyes.

### Calculation of IOL tilt and decentration

The computational procedure to estimate the tilt and decentration from the OCT images is shown in Figure 1. The details about it just as follows:

1) Calculate the zoom factor of eye cross-section image and plane graph.

The zoom factor 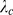 of cross-section image can be calculated using the real distance and image distance of scale shown in Figure 2.
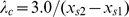



The real x direction distance 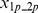 of boundary of pupil in the cross-section image is 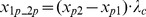



Then, through the corresponding relation of the point 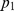 to 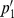 and 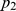 to 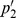 in the cross-section image and plane graph, the zoom factor 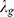 of plane graph can be calculated
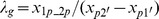



2) obtain the center coordinate and radius of sphere that contain the anterior/posterior surface of intraocular lens respectively

For obtaining the center and radius of sphere that contain the anterior/posterior surface of intraocular lens respectively, the three dimensional coordinate of four points on the anterior and posterior surface should be obtained.

Before getting the coordinates of these points, the reference point should be chosen. Through the analysis of different images, the point of intersection of different cross-section plane in the plane graph was used as the Reference point in plane graph. The point of intersection of correspond location with the iris was used as the Reference point in cross-section plane shown in Figure 1.

The step that obtaining the Reference point as following:

Firstly, obtain the x and y coordinates of reference point in the plane graph, that is,
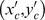



Then, using the x coordinate of reference point and point 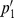, obtain the real distance of these two points in the plane graph. Through the scale factor of cross-section plane, obtain the x coordinate of reference point in the cross-section plane,
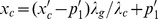



Through the point of intersection of location of reference point and iris, the z coordinate of the reference point will be obtained. The three dimensional coordinate of reference point is,
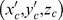



For obtaining the three dimensional coordinate of arbitrary point on the anterior/posterior surface of intraocular lens respectively, we choose two section planes, that is 0–180degree and 90–270degree cross-section planes (Figure 2). The four points on the anterior/posterior surface were chosen on these section planes and obtain the coordinate of these points. These coordinate can be used to construct the sphere that contain the anterior/posterior surface.

The step of obtaining the coordinate of arbitrary point relative to the reference point 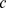 is:

Chose a point on the cross-section plane line in the plane graph for example 0–180degree section plane, obtain the x and y coordinate, 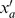,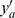. Then, obtain the location of this point on the cross-section plane,
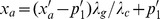



Then, obtain the z coordinate of this point 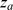 on the surface of intraocular lens, the three dimensional real coordinate of this point relative to reference point is,
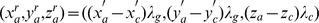



Using these steps, the coordinate of another point can be obtained.

Using the three dimensional coordinate of arbitrary four points on the anterior/posterior surface, the center coordinate and radius of sphere one sphere can be obtained.

The center coordinate (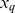,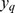,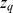) and radius 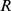
of sphere that contain the anterior/posterior surface of intraocular lens can be obtained through geometry relation using the coordinate of arbitrary four points respectively.

3) The three dimension coordinate of center and normal of intraocular lens

Through the relation of two intersection sphere (Figure 3), the center coordinates (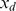,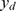,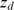), radius and normal of intraocular lens can be obtained.

Based on the geometry relation shown in Figure 3, we can obtain that,
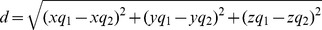


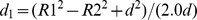



The coordinate of center of intersection circle of two spheres (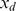,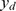,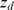), that is the center of lens point D,



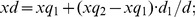





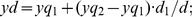


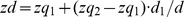



The normal (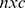,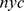,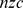) of this intersection circle is



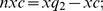





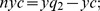


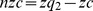



4) The three dimension coordinate and normal of pupil

Based on the coordinate of three point on the boundary of pupil, the three dimension coordinate (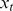,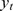,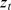), the radius 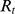
and normal of pupil (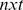,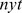,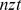) can be obtained through the geometry relation of three point determining a circle.

5) The decentration and tilt angle of intraocular lens

The center of intraocular lens is (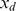,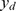,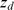), the center of pupil is (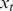,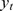,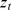), so the decentration of intraocular lens relative to the pupil is
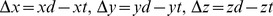



The spatial distance of decentration is
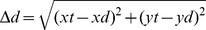



The tilt angle 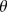 (unit: deg) between the IOL plane and the papillary plane is 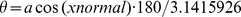



Where,
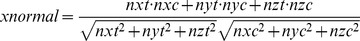



### Subjects

After image quality control for original fifty-nine eyes of 59 patients, thirty-nine eyes of 39 patients, who were diagnosed with age-related cataract and had undergone an uneventful phacoemulsification with the IOL in the capsular bag, were included in this study finally. The study protocol adheres to the tenets of the Declaration of Helsinki. Ethics committee approval was obtained from the Shanghai Clinical Research Center and informed consent was obtained from all subjects who participate in the study. To avoid the possibility of changes in the alignment of the IOLs over time, the patients' postoperative clinical evaluations at 12 months or more were considered in our study. The inclusion criterion was the receipt of an in-the-bag fixated IOL after uneventful phacoemulsification, and the exclusion criterion was complicated cataract surgery with posterior capsular rent or yttrium-aluminum-garner capsulotomy [10].

### Statistical analysis

The data were analyzed by SPSS software version 13.0 (SPSS, Inc., Chicago, Illinois, USA). To quantify the reproducibility of repeated measurements performed by the same observer at different time point and by different observers, we calculated the repeatability, reproducibility, and intraclass correlation coefficients (ICCs). We used general linear model of repeated measurement to analyze the repeatability of the method. A Pearson correlation was conducted to analyze the relationship between every two indexes including ORA, BCVA, total astigmatism, tilted angle and decentration. All the tests had a significance level of 5%.

## Results

Thirty-nine eyes were evaluated in this study. The mean period between the surgery date and the OCT imaging date was 14±1.9 months. The mean IOL power was 20.5±2.87 Diopters (D) and the IOL thickness was 1.24±0.29 mm. The mean postoperative total astigmatism and BCVA were 1.14±0.48 D and 0.85±0.12, respectively. The corneal astigmatism resulting from Atlas corneal topography was 0.68±0.33 D. The mean ORA was 0.48±0.30 D. The mean angle (θ) between the pupillary plane and the IOL plane was 2.94±0.99 degrees. The mean IOL decentrations of dx and dy were 0.32±0.26 mm and 0.40±0.27 mm, respectively. The ds of the IOL decentration was 0.56±0.31 mm (Table 1, 2).

**Table 1:** Anterior segment optical coherence tomography estimation of in-the-bag intraocular lens tilt and decentration in relation to the pupillary plane.

	Mean ± SD (n = 39)
X-axis distance	0.32±0.26
Y-axis distance	0.40±0.27
Spatial distance	0.56±0.31
*Angle (in degrees)	2.94±0.99

Note: * with reference to the pupillary plane; SD  =  standard deviation.

**Table 2:** Best-corrected visual acuity and astigmatism values of subjects.

	Mean ± SD (n = 39)
BCVA	0.87±0.13
Total astigmatism (D)	1.14±0.48
Corneal astigmatism (D)	0.68±0.33
ORA (D)	0.48±0.30

Note: BCVA  =  best-corrected visual acuity; ORA  =  ocular residual astigmatism; D  =  diopters; SD  =  standard deviation.

### Interoperator reproducibility

**Figure 7:**
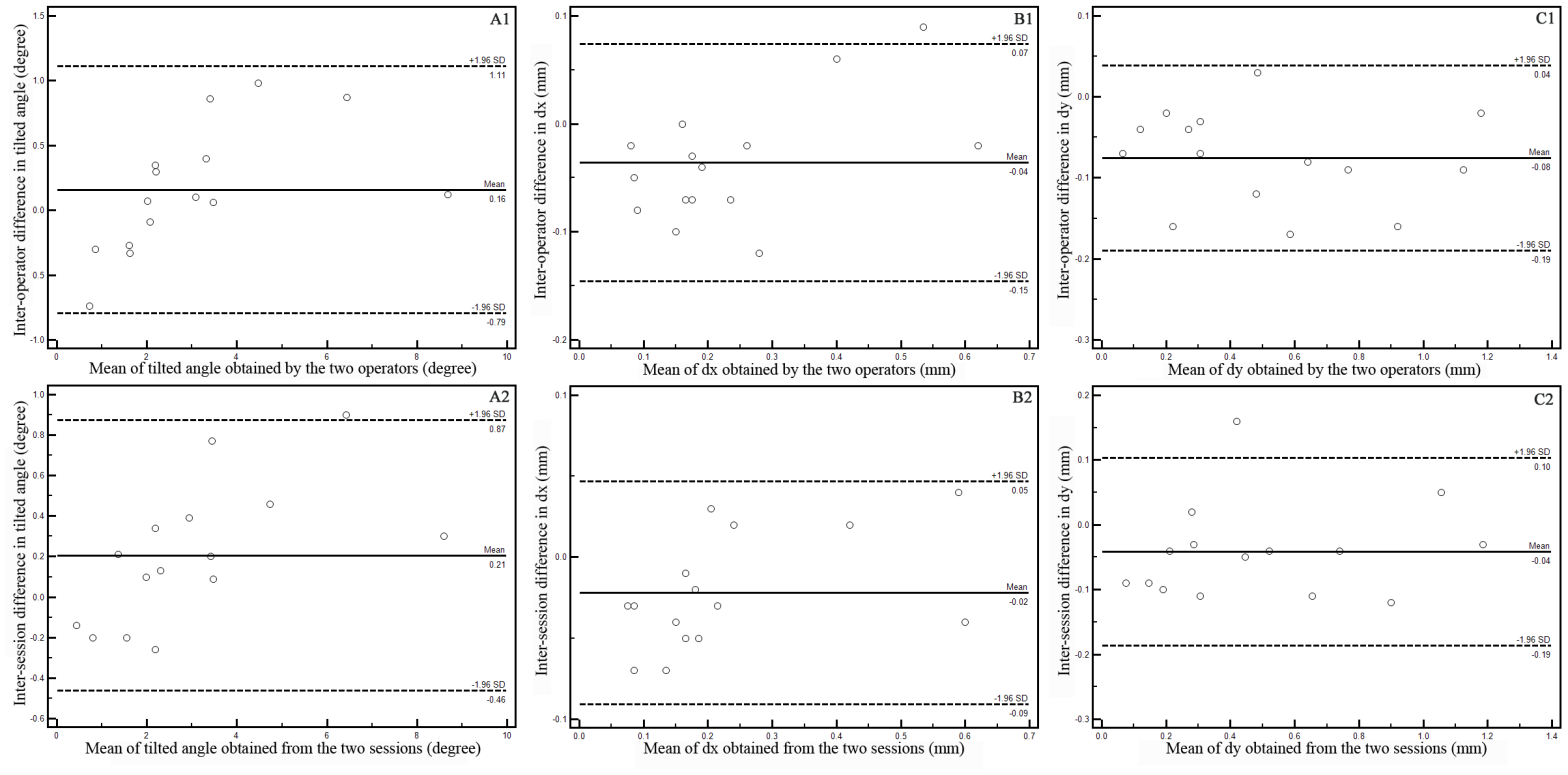
Graph of data from inter-operator (A1, B1, C1) and inter-session (A2, B2, C2) reproducibility study (n = 15).

In 15 randomly chosen subjects, the overall average tilted angle and decentration distance were measured by operator XG.W and J.D (Table 3). Table 4 shows the intraclass correlation coefficient (ICC) for interoperator and intersession reproducibility and they were all around 0.95. Figure 7 (A1, B1, C1) shows the graph of differences against means of the average tilted angle and decentration distance. The 95% limits of agreement (LoA), defined as mean interoperator difference in tilted angle/decentration distance ±(1.96 SD of differnence), was shown in each graph.

**Table 3:** The tilted angle and decentration distance of different measurements.

	Measurements, Mean ± SD (n = 15)		
	Measurement 1	Measurement 2	Measurement 3
X-axis distance	0.22±0.18	0.24±0.16	0.26±0.15
Y-axis distance	0.47±0.35	0.51±0.34	0.55±0.36
*Angle (in degrees)	3.16±2.28	2.95±2.07	3.0±2.0

Note: Measurement 1 and 2 indicate the 2 measurements performed by observer XG.W on two sessions; measurement 3, the third measurement performed by observer J.D. * with reference to the pupillary plane; SD  =  standard deviation.

**Table 4:** Interoperator and intersession reproducibility analysis of IOL tilted angle and decentration.

	Intraclass Correlation	95% Confidence Interval	
		Lower Bound	Upper Bound
Angle 1-2	0.984	0.955	0.995
Angle 1-3	0.974	0.925	0.991
Dx 1-2	0.972	0.920	0.990
Dx 1-3	0.941	0.834	0.980
Dy 1-2	0.971	0.920	0.990
Dy 1-3	0.987	0.960	0.995

Note: Number 1 and 2 indicate the 2 measurements performed by observer XG.W on two sessions; measurement 3, the third measurement performed by observer J.D. Dx  =  decentration distance in X-axis, Dy  =  decentration distance in Y-axis.

### Intersession Reproducibility

Intersession reproducibility was investigated in a similar way. From the graph of differences against mean for the intersession data (Figure 7: A2, B2, C2), it can be seen that almost 100% of the values fall within 1.96 SDs of the mean.

### Repeatability

**Figure 8:**
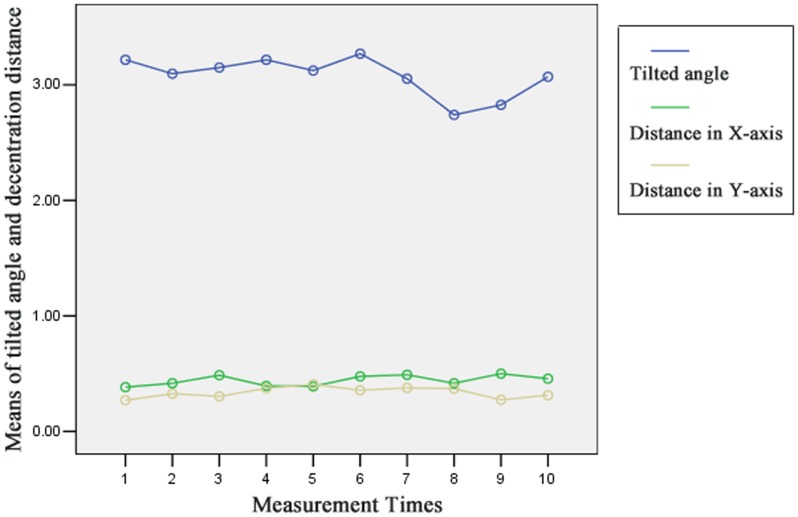
Graph of data from repeatability study (n = 3).

First, we established the general linear model with indexes (tilted angle, dx and dy) as between-subject factors and with measurement time as within-subject factor in order to perform repeated measurements. The data showed that measurement times did not meet the Mauchly sphericity null hypothesis (P<0.001). So we chose the Greenhouse-Geisser results and it showed that there was no statistical significance in measurement times (F = 1.030, P = 0.393), and Figure 8 showed that there was no statistical difference of the interaction between measurement times and the indexes, too (F = 1.216, P = 0.351). In other words, it showed good repeatability in the test of the indexes measurement.

### Correlations between indexes

**Figure 9:**
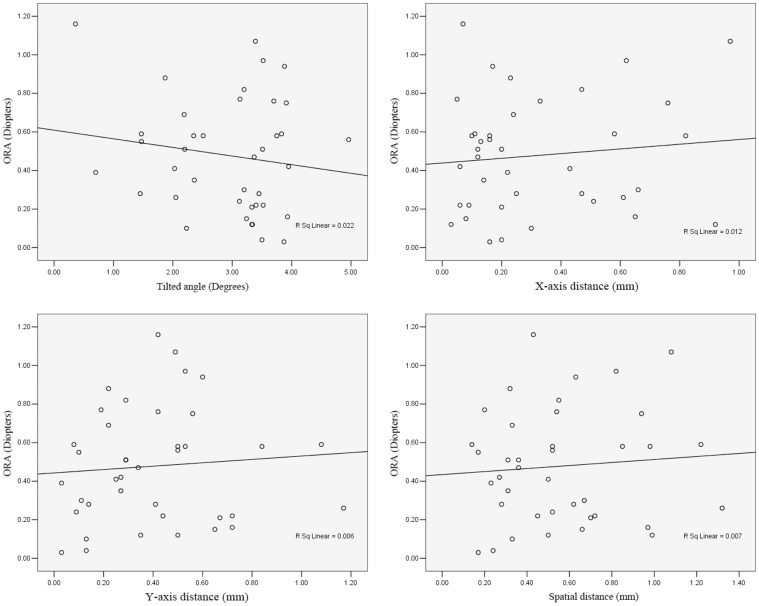
Scatterplot showing the correlation of the ocular residual astigmatism (ORA) versus the tilted angle, decentration of the x-axis, the y-axis and the spatial distance of decentration.

There was no significant correlation between the total astigmatism and the tilted angle (θ; r = −0.103; *P* = 0.532) or the decentration distance (dx: r = −0.054, *P* = 0.743; dy: r = 0.015, *P* = 0.926; ds: r = −0.023, *P* = 0.891). Similarly, there was no significant correlation between the ORA and the tilted angle (θ; r = −0.149; *P* = 0.365) or the decentration distance (dx: r = 0.109, *P* = 0.511; dy: r = 0.080, *P* = 0.629; ds: r = 0.081, *P* = 0.625), as shown in Figure 9.

**Figure 10:**
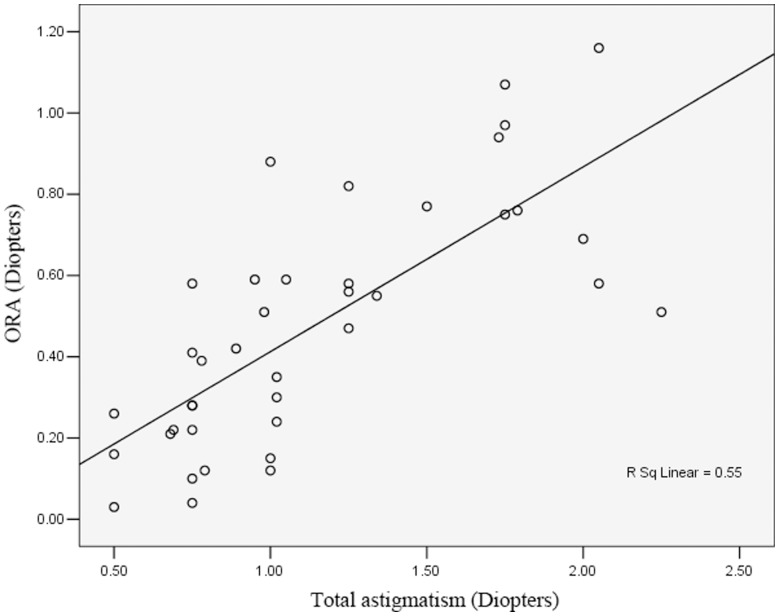
Scatterplot showing the correlation between the ocular residual astigmatism (ORA) and the total astigmatism in diopters; y-axis  =  ORA in diopters.

However, there was a significant correlation of ORA with total astigmatism (Figure 10; r = 0.742, *P*<0.001). There was no significant correlation between postoperative BCVA and ORA (r = 0.156; *P* = 0.344), total astigmatism (r = 0.012; *P* = 0.942), tilted angle (θ; r = 0.172; *P* = 0.295), or decentration distance (dx: r = 0.191, *P* = 0.244; dy: r = 0.253, *P* = 0.121; ds: r = 0.298, *P* = 0.065).

## Discussion

As a convenient, noninvasive and high-resolution imaging method, AS-OCT can provide cross-sectional tomography of the anterior ocular structure *in vivo*. This method shows great advantages in the diagnosis of glaucoma, anterior segment tumors, cataracts and refractive surgery follow-up [13]–[16]. Since 1988, Philips *et al*. [17] reported an average IOL tilt of 7.8 degrees and a mean decentration of 0.7 mm with PMMA IOLs using the Purkinje imaging system, many researchers have been concerned about the IOL displacement, which has been noticeably minimized due to important advancements in surgery skills and IOL designs. The precise evaluation of the malposition of IOL is important for the follow-up after cataract surgery because it may cause refractive errors and retinal image problems, which may worsen visual acuity [18], [19], and it may give some indications for exchange, repositioning, or removal of a posterior chamber IOL because it can be performed immediately in the postoperative period, even in eyes with bad corneal clarity due to edema. Therefore, we described the application of AS-OCT to analyze the IOL decentration and tilt by a 3D-reconstruction method.

Using the Scheimpflug photography system, Baumeister *et al.* [20] found that the postoperative IOLs showed a relatively stable position regarding tilt and decentration in the first 12 postoperative months regardless of the material and edge design used. However, to avoid possible changes in the alignment of the IOLs over time, only the patients, who were seen over 12 postoperative months, were included in this the study. At the same time, we chose 5-minute dark adaptation to replace the pupil dilation by mydriatic to minimize the potential influence of ciliary muscle accommodation.

In this study, we proposed a 3D-reconstruction method to calculate the IOL tilt and decentration using a commercial OCT imaging system, which is different from the Purkinje or Scheimpflug imaging system and ultrasound biomicroscopy (UBM) [6], [11], [21]. The Purkinje imaging system has limitations when lenses are very flat, and it also relies on the proper measurement of the anterior and posterior lens radius of curvature. The Scheimpflug system requires sufficient pupil dilation, which we did not use because pupil dilation may influence the IOL position due to ciliary muscle accommodation, and the pupil size after dark adaptation can meet the requirements for AS-OCT image capture, making the posterior IOL surface visible. The AS-OCT can be performed in the early postoperative period because the coupling fluid application is not necessary compared with UBM. Other advantages include the noninvasive nature of the procedure, the ability to obtain high-resolution images, and the fact that manipulation is easy and fast. However, UBM can image the haptic position below the iris, which is not possible using AS-OCT.

Considering using the iris pigment epithelium layer as reference layer for IOL evaluation in Loya *et al* study [22] by UBM and the limitation of penetrating depth for OCT technology, the iris smooth muscle can be recognized more easily by an observer than other structures such as the Schlemm canal, the trabecular meshwork, iris pigment epithelium layer and the limbus, with the current time-domain OCT system. Therefore, we used this plane as the reference plane to evaluate the tilt of the IOL, which is different from the Kumar *et al.* [10] study using the limbus as a reference line. The tilted angle was similar in some previous studies conducted with the Scheimpflug system [23], [24], but it was larger than 1.52±0.9 degrees in the Kumar study [10]. We deduced that the difference resulted from not only the different reference line/plane, but also the different method of calculation. It is well known that the IOL has in-the-bag tilt and decentration, but the OCT scan light is parallel with the ground. In this condition, if we just analyze the angle between the reference line and one line in the IOL plane but not the real angle between the two planes, the angle will be different when different angular single scan line models are used, and it may even appear to be parallel at a specific scan angle. Therefore, the method of Kumar *et al*. [10] may underestimate the real angle between the two planes. The 3D-reconstrction method, which can calculate the real angle between the two planes rather than two lines, is more precise. Moreover, this method can be used with less than perfect images of the anterior chamber angle at every angle of the quadrant-scan model, and it can be used with imperfect images such as the images at 90–270 degrees in Figure 5 if the pupil and IOL images are suitable.

Another aspect of IOL misalignment is the decentration, which occurs even after an uneventful implantation [25]. The incidence of IOL misalignment has been substantially reduced due to the improvement surgical techniques and IOL designs, but the reports of extreme IOL misalignment requiring explanation existed all the time [26], [27]. According to the study by Koryna *et al.* [28], the refractive effect of IOL displacement depends on the magnitude of the tilt and decentration. Moreover, more than 5 degrees of tilt and greater than 1 mm of decentration can cause relevant myopic shift and oblique astigmatism, respectively. After the 3D-reconstruction, there were three types of decentration distance (dx, dy, ds) to be analyzed in this study. We did not do statistical analysis for the distance in the z-axis, which was got by this method. Because the reference plane is just a corresponding reference and the z-axis values could not give better clinical application compared with dx and dy. Wang *et al*. [23], who used the Scheimpflug photographic technique, reported mean decentration values of 0.30±0.17 mm and 0.34±0.20 mm for IOLs of different materials, which were similar to the dx in our results but less than dy and ds. Other previous studies reported similar results as well [24], [29].

There are case reports describing major tilting that has resulted in decreased visual function, but the majority of researchers found that the clinical relevance of tilt and decentration was limited, which is consistent with our results [28], [30].

The single-line scan model provided insufficient information for 3D-reconstruction analysis; accordingly, we chose the quadrant-scan model. This model took longer than the single-line scan model. Therefore, several potential factors in AS-OCT image capturing may affect the tilt and decentration values, such as the lack of an eye-tracking technique, the lower scan speed, the relatively longer capture time, the lack of a real-time capture technique for the eye plane image and the cross-sectional image and fixation fluctuation of the patient. However, the 3-D analysis methodology for assessing the tilt and decentration of IOL in this study is an innovative method that may play an important role in the development of AS-OCT technology in future.

In our study, we only selected the spherical IOL patients because the anterior and posterior surface of the spherical IOL is easy to do 3D-reconstruction analysis to test this method. The aspherical IOL is widely used, and we plan to develop a better way to perform the analysis under this condition in the future. The method of the mathematical model is totally accurate, so we do not need to test the accuracy of it. We chose the iris smooth muscle as different reference plane compared with limbus to finish the whole process and there is still a big debate on where to center the IOL. This tissue is well recognized on OCT image, but it has limitations for eyes with aniridia or abnormal configuration. Although the visual axis is little closer to the Purkinje reflex than the pupil center, we got similar results of tilted angle and decentration in our study. At the same time, we provide another method to analyze the IOL malposition, and we will compare different reference plane in the next step. We only chose good phacoemulsification case for our analysis, so it is a good result for us that there was no significant correlation between clinical parameters and decentration/tilt values. Moreover, additional studies may be needed to compare several methods of IOL position analysis, including Purkinje images, Scheimpflug images and OCT.

## Conclusions

An uneventful in-the-bag IOL maintains a decentration of less than 1 mm and an angle of less than 4 degrees with reference to the iris smooth muscle plane according to AS-OCT, which did not influence the astigmatism or the BCVA in this study. The measurement of tilted angle and decenrtation distance have been showed to be both repeatable and reproducible and this indicate that the AS-OCT can be used as an alternative for the analysis of IOL tilt and decentration by the method of 3D-reconstruction.
